# Nutritional inequalities among under-five children: a geospatial analysis of hotspots and cold spots in 73 low- and middle-income countries

**DOI:** 10.1186/s12939-022-01733-1

**Published:** 2022-09-15

**Authors:** Rafi Amir-ud-Din, Sakina Fawad, Lubna Naz, Sameen Zafar, Ramesh Kumar, Sathirakorn Pongpanich

**Affiliations:** 1 Department of Economics, COMSATS University Islamabad, Lahore Campus, Lahore, Pakistan; 2 Department of Economics, Institute of Business Administration, Karachi, Pakistan; 3 Suleman Dawood School of Business, Lahore University of Management Sciences (LUMS), Lahore, Pakistan; 4 Health Services Academy, Ministry of NHSR & C, Government of Pakistan, Islamabad, Pakistan; 5 College of Public Health Sciences, Chulalongkorn University, Bangkok, 10330 Thailand

**Keywords:** Stunting, Wasting, Underweight, Inequality, LISA, Moran’s *I*

## Abstract

**Background:**

Child undernutrition is a severe health problem in the developing world, which affects children’s development in the long term. This study analyses the extent and patterns of under-five child undernutrition using Demographic and Health Surveys (DHS) for 73 low- and middle-income countries (LMICs).

**Methods:**

First, we mapped the prevalence of undernutrition in the developing world. Second, using the LISA (a local indicator of spatial association) technique, we analyzed the geographical patterns in undernutrition to highlight the localized hotspots (regions with high undernutrition prevalence surrounded by similar other regions), cold spots (regions with low undernutrition prevalence surrounded by similar other regions), and outliers (regions with high undernutrition surrounded by low undernutrition and vice versa). Third, we used Moran’s *I* to find global patterns in child undernutrition.

**Results:**

We find that South Asia has the highest under-five child undernutrition rates. The intra-country nutritional inequalities are highest in Burundi (stunting), Kenya (wasting), and Madagascar (underweight). The local indicator of spatial association (LISA) analysis suggests that South Asia, Middle East and North Africa (MENA) region, and Sub-Saharan Africa are undernutrition hotspots and Europe and Central Asia and Latin America, and the Caribbean are undernutrition cold spots (regions with low undernutrition surrounded by similar other regions). Getis Ord-Gi* estimates generally support LISA analysis. Moran’s *I* and Geary’s *C* gave similar results about the global patterns of undernutrition. Geographically weighted regressions suggest that several socioeconomic indicators significantly explain child undernutrition.

**Conclusions:**

We found a significant within and across country variation in stunting, wasting and underweight rates among the under-five children’s population. The geospatial analysis also suggested that stunting, wasting, and underweight patterns exhibit clear regional patterns, underscoring the need for coordinated interventions at the regional level.

## Introduction

Child undernutrition is a severe health problem in the developing world, affecting children’s development in the long term. In 2020, almost 149 million under-five children were stunted, 59 million were wasted, and 85 million were moderately or severely underweight [1]. In 2016 alone, childhood undernutrition, i.e., stunting, wasting, and underweight, caused one million deaths globally [2]. Child undernutrition shows significant variations across and within national boundaries. In 2017, the share of undernourished under-five children was 20.4% in Africa, 11.4% in some regions of Asia, and 6.1% in Latin America [3].

A wide range of studies has analyzed the factors contributing to child undernutrition. For instance, a study in the Middle East and North Africa found that characteristics like a child’s age, gender, birth order, and twinning were significant determinants of child undernutrition [4]. Another study revealed that undernutrition results from disorders in nutrient assimilation and defects in the immune system, which are generally associated with the nutritional status of parents [5]. Some other predictors of child undernutrition include household wealth, the educational and nutritional profile of the mother, food security, geography, and sewerage conditions [6]. Nutritional supplements consumed by the mother, such as folic acid, vitamins, and minerals, may also explain nutritional differences among children [7]. In some countries like India, gender biases are deep-rooted and lead to disproportionately worse nutritional outcomes for women and children [8]. In addition, children from poor households bear a disproportionately larger burden of malnourishment than rich households [9].

Undernutrition has lasting adverse consequences. Early childhood stunting is associated with poor cognitive, motor, linguistic, and socio-emotional skills [10], low IQ and undesirable educational outcomes [11], reduced enthusiasm and increased apathy [12], pregnancy-related complications, and birth to smaller babies among women in their later life [13]. Childhood wasting increases the risk of lower respiratory infection-related under-5 mortality and is associated with a rise in blood pressure and poor educational outcomes [14]. Childhood underweight is associated with a compromised immune system, increasing the risk of infections, higher risk of fractures and osteoporosis, and fertility-related issues such as failed conception and pregnancy-related problems, and hormonal imbalance and menstruation problems [15].

Reducing health-related inequalities is an essential component of the sustainable development goals (SDGs). While the incidence of various types of child undernutrition has been well-established in the existing literature, few studies have estimated the geographic distribution of child malnourishment in developing economies. One consequence of incomplete information regarding the geographic distribution of undernutrition is that policy interventions may not generally be targeted and may be concentrated in the areas which require the least intervention.

This study aims to understand the geographic patterns of child undernutrition using various geospatial techniques within and across 73 low- and middle-income countries (LMICs) using DHS surveys to provide policy insights for targeted interventions to reduce child malnourishment. The significant contribution of this study is that it employs a large sample of low- and middle-income countries with sub-regional level information for three indicators of under-5 child undernutrition, including stunting, wasting, and underweight.

### Theoretical reflections on health inequalities

Different theories have been applied in the context of health inequalities. In a detailed account[16], present four arguments to establish that health inequalities are fundamentally undesirable, and their reduction must be prioritized in public policy. First, inequalities are unfair since poor health is the consequence of an unjust distribution of the underlying social determinants of health. Second, inequalities affect everyone since some forms of health inequalities have externalities on the rest of society. Third, inequalities are avoidable. Health disparities are preventable to the extent that they emanate from identifiable policy options exercised by governments. Therefore, a government that cares about improving the population’s health should incorporate the reduction of health inequalities as part of its policy agenda. Fourth, interventions to reduce health inequalities are cost-effective. Public health programs that reduce health inequalities can be cost-effective. A case can be made to prioritize such programs as providing cervical cancer screening to poor women on efficiency grounds[16]. conclude that fairness is likely the most compelling argument favoring action to reduce health disparities. There is persuasive evidence for some outcomes that reducing inequalities will diminish adverse spill-over effects on the health of society at large.

**Fig. 1:**
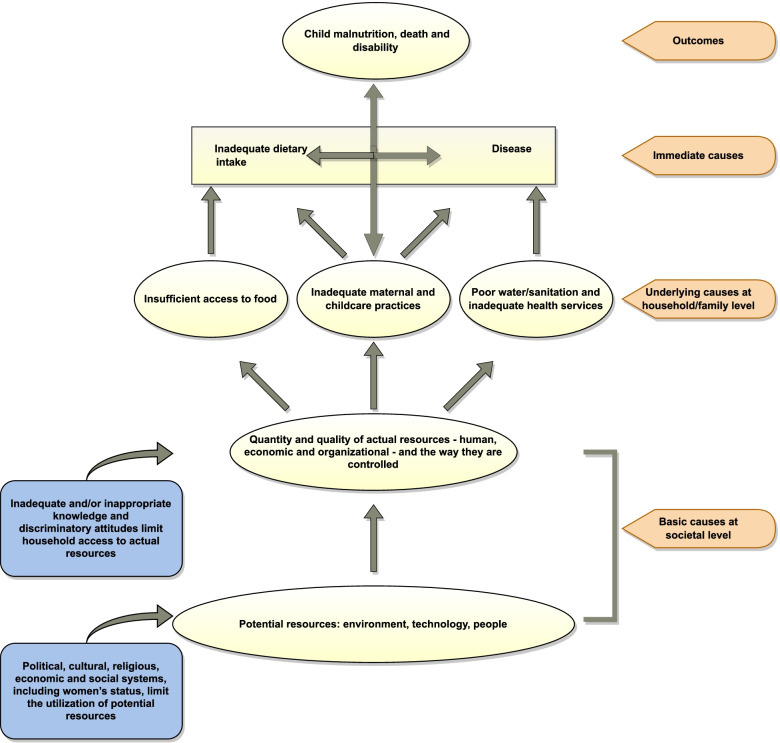
Theoretical framework. Source: UNICEF (1998) [18]

What causes undernutrition among children has received significant scholarly attention in the past several decades. UNICEF came up with a comprehensive conceptual framework in 1998 to explain the reasons behind child malnutrition [17]. UNICEF conceptual framework partitioned causes behind child malnutrition into three categories, namely, basic, underlying, and immediate (Fig. 1). UNICEF conceptual framework explained that basic causes were specific to the societal level. Socioeconomic and religio-cultural, and political processes at a societal level limit the utilization of human, technological, and environmental resources. Inadequate knowledge and discriminatory attitudes at the household level limit access to food, effective childcare practices, and water/sanitation and health services, and make up the underlying causes of child malnutrition. The underlying causes, in turn, lead to immediate causes of child malnutrition and may take the form of inadequate dietary intake and diseases, ultimately resulting in child malnutrition.

More recent studies illustrate that rising social inequality and poverty, greater job insecurity, rising unemployment, and privatization of public goods and services make the inequalities in health more severe [19, 20]. Individual-level inequalities in health in developed economies like the UK and USA have also been explained by factors including income, education, gender, race/ethnicity, and immigration status [21, 22]. A close examination of the relationship between aggregate indicators of income inequality and aggregate health outcomes across the industrialized nations suggests that income inequality is considerably associated with the population-health indicators such as life expectancy and infant mortality [21, 23].

Almost all modern societies establish systems for allocating resources and institutional mechanisms for transforming social and individual resources into health. Still, vast health inequalities exist across societies. According to the fundamental-cause theory proposed by [24], socioeconomic status serves as the ‘fundamental cause of the disease’ because it indicates the degree of access to the resources essential to avoid disease and their effects and defines the susceptibility to risk factors of the disease and their outcomes. Consequently, the mortality-reducing technologies and knowledge steepen the health gradient because the better-off can benefit from their resources while leaving the poor people worse-off [25].

Traditionally, most Western models of health viewed sickness and disease as a product of individual factors such as personal behaviors and genetic predisposition. Consequently, healthcare interventions focused primarily on fixing the individual rather than on external factors.

The World Health Organization’s (WHO) Social Determinants of Health (SDH) framework adopts a much broader ecological perspective to incorporate interventions at multiple levels to achieve health objectives on an equitable basis. According to the WHO, health and well-being disparities are attributable to the circumstances in which “people are born, grow, live, work, and age” [26]. The SDH framework includes policies, education, and living conditions [27]. Within each society, those who are powerful and own resources determine which exact circumstances are valued and which are not. Typically, the decisions made by power wielders protect the values and well-being of themselves and their constituents. Meanwhile, the values of the marginalized populations tend to suffer across several domains, adversely impacting their health. Thus, personal health is influenced not only by genetics and lifestyle but also by systemic social inequities [28].

Some do not perceive health inequalities as the direct result of the income inequality approach to health inequalities. They try to understand health inequalities within the framework of the class-based model by concentrating on both the causes and the consequences of income inequalities. In such a model, the relationship between income inequality and health appears as a special case within a broader causal chain. It is argued that the global and national socio-political and economic trends have increased the power of business classes at the cost of the working classes. The neo-liberal policies accompanying these trends led to increased income inequality which, in turn, increased inequalities in access to many other health-relevant resources. But international pressures toward neo-liberal doctrines and policies are differentially resisted by several nations because of the historically entrenched variations in class and institutional structures [29].

Health inequalities have also been explained by the difference in the relative and absolute income differentials, where the link between per capita income and health status shows a clear gradient [30] argued that Gross National Product (GNP) per capita is the most important correlate of the average levels of the health status amongst developing nations. GNP per capita above $5,000–$10,000 reflects mainly the range at which chronic diseases begin to displace acute illness as the chief cause of death[30]. contends that the degree of income inequality, rather than national wealth, is the most important basis of national differences in health status. Even below the $5,000–$10,000 mark, there is a wide variation in health for any health, particularly GNP per capita level. Hence, the discussion of the developing countries’ health cannot be dismissed as the simple product of GNP per capita [31].

Social hierarchies are also believed to produce disease because of the poor self-esteem associated with lower status, negatively influencing health through psycho-neuro-biological pathways [30]. A study in England established that high-level British civil servants showed poorer health than those even greater in the hierarchy, suggesting that relative rather than absolute income differences motivate the relationships between income and health [29].

### Spatial analysis of malnutrition

Spatial analysis is being increasingly used to analyze child malnutrition in recent years. A study in Ethiopia used a geo-additive mixed model via Nested Laplace Approximation to analyze child malnutrition [32]. Another study in Ethiopia studied the double and triple burden of malnutrition using spatial and survey regression analysis, where spatial analysis consisted of Moran’s *I* statistic and inverse distance weighted interpolation [33]. A spatial analysis using Moran’s *I* and LISA statistics was done to identify local occurrences of the factors contributing to child undernutrition in India [34]. A study used spatial lag regression to identify climatic determinants of malnutrition in Bangladesh [35]. A study in Peru conducted a district-level spatial analysis using Moran’s global and local indices to analyze childhood malnutrition [36].

## Materials and methods

### Data

This study aims to highlight geographic disparities in the indicators of under-five child undernutrition in the developing world. The data is obtained from the Demographic and Health Surveys (DHS). Our study used data from a sample of 73 countries and 699 sub-regions. Currently, DHS provides information for only 73 countries. DHS is a large and standardized household survey [37]. The targeted sample of each country is based on nationally representative sampling plans. The surveys collect data on indicators of access to maternal and child health interventions, illness, treatment, and nutritional status. These surveys provide important information on the nutritional status of young children across countries for a wide range of indicators. Besides, these surveys also gather a comprehensive set of standardized socioeconomic indicators and other similar information. The units of investigation utilized for our analysis are DHS regions and sub-regions. We used the standard definitions of three measures of under-five child undernutrition as presented in Table 1. A child is considered stunted, wasted, or underweight if her z-score is 2 standard deviations below the mean z-score on the WHO Child Growth Standards [38]. DHS measures stunting (wasting and underweight) in two different ways, that is, z-score < -2 (moderately stunted) and z-score < -3 (severely stunted), and our definition includes both moderately stunted and severely stunted (wasted and underweight).

**Table 1:** Measurement of the under-5 child malnutrition

Indicators	Definitions
Stunting	Children whose height-for-age z- score is is below minus 2 (-2.0) standard deviations (SD) below the mean on the WHO Child Growth Standards
Wasting	Children whose weight-for-height z-score is is below minus 2 (-2.0) standard deviations (SD) below the mean on the WHO Child Growth Standards
Underweight	Children whose weight-for-age z-score is is below minus 2 (-2.0) standard deviations (SD) below the mean on the WHO Child Growth Standards

Demographic and Health Surveys

### Empirical analysis

We use the DHS data to analyze the indicators of stunting, wasting, and being underweight among under-five children in 73 developing economies and 699 sub-regions. DHS data are collected repeatedly over time. Thus, we selected the most recent DHS wave when multiple DHS data waves were available.

There is substantial evidence to suggest that wide within-country disparities in the status of child undernutrition exist [37]. We give a country-level *spectrum of child undernutrition*, with the lowest and highest levels of undernutrition in a country and the national rate of stunting, wasting, and underweight. As an illustration, in Pakistan’s case, we chose the province (the DHS sub-region) with the lowest stunting rates and the province with the highest stunting rates, and the average national stunting rate. The national stunting rate, by construction, is between the highest and lowest stunting rates.

### Geospatial analysis

We used the DHS survey data to compute under-five child undernutrition in three variables: stunting, wasting, and underweight. To map data to geographical regions, we used the Spatial Data Repository data [39] and matched indicator estimates to the geospatial data using ArcGIS Pro 2.8.3.

We used the exploratory spatial data analysis (ESDA) technique to analyze child undernutrition in the selected countries. ESDA visualizes and measures the spatial autocorrelation between and among regions. We initially assessed the spatial trends of child undernutrition measures, both inside and across the national boundaries.

Then we identified the areas with statistically significant clustering of hotspots (high levels of undernutrition) and cold spots (low levels of undernutrition). The latter analysis gave the intra-and within-country information about the high and low-performing areas concerning geographic proximity [40].

### Spatial autocorrelation of child undernutrition indicators

We first constructed thematic maps of childhood undernutrition in ArcGIS [41] to assess child undernutrition prevalence, both inside and across national boundaries. The contiguous areas are more likely to be similar than those away from each other. Therefore, the spatial autocorrelation measures the degree to which a sub-region is similar or dissimilar to the neighboring sub-regions for a specific indicator of undernutrition [42]. Given the study sample, spatial autocorrelation can be conducted globally or at the local level. The global measure summarises the spatial autocorrelation over the entire study area. In contrast, the local measure analyses localized spatial autocorrelation inside the study area [43].

Moran’s *I* statistic was used to measure global spatial autocorrelation or clustering of undernutrition [44]. The Moran’s *I* test result ranges from -1 to 1, where a value near 0 shows no statistically significant spatial clustering over the study area. The other possibilities are given in the following equation.

Moran’s *I* measurement was initially utilized to quantify worldwide spatial autocorrelation of ailing health [44].

$${\mathrm{Moran}}^{\mathrm{^{\prime}}}\mathrm{s} I= \left\{\begin{array}{c}<0\\ 0\\ >0\end{array}\right. \begin{array}{c}\mathrm{Spatial clustering}:\mathrm{ neighboring regions have different values}\\ \mathrm{No significant spatial clustering }\\ \mathrm{Spatial clustering}:\mathrm{ neighboring regions have similar values}\end{array}$$

We also used another spatial autocorrelation analysis tool, Geary’s C, to understand the extent of spatial clustering in under-5 undernutrition. Geary’s C can vary from 0 to 2, where 1 indicates no spatial autocorrelation, values close to 0 indicate positive spatial autocorrelation, and values close to 2 indicate negative autocorrelation [45]. We used SpaceStat Version 4.0.21 to estimate Geary’s *C*.

To measure localized spatial autocorrelation of child undernutrition, a local indicator of spatial association (LISA) for Moran’s *I* was used, which indicated if significant spatial clusters per each location in the sample region exist or do not exist [46]. The LISA analysis produces a spatial layer that gives five different types of spatial associations and potential outliers [47].

- Not significant: the areas which do not exhibit any statistically significant spatial autocorrelation ($p \le 0.05)$.
- High-high: this type of spatial association demonstrates that the high values are encompassed by the other neighboring high values. It must be noted that the high values are not high in absolute terms but in relative terms. In this kind of association, the geographical regions with high rates of prevalence of child undernutrition are surrounded by other geographical regions with similarly high rates
- Low-low: the low values are surrounded by other low values. In this form of association, the geographical regions with low prevalence rates of child undernutrition are surrounded by similar geographical areas with a low prevalence of child undernutrition.
- Low – high: the low values are surrounded by high values. In other words, the areas with a low incidence of child undernutrition are surrounded by other sub-regions with high prevalence rates of child undernutrition. The geographical areas with this type of association reveal nutritional inequality and offer important insights regarding the factors contributing to the low undernutrition levels even when the neighboring regions have high undernutrition levels. Understanding how one region could achieve a lower undernutrition level compared to another region is essential knowledge from the perspective of policy formulations.
- High-low: the high values are surrounded by low values. It means that the regions which have high prevalence rates of child undernutrition are surrounded by other sub-regions with low levels of child undernutrition. The geographical areas with this type of association again reflect nutritional inequality and provide insights about the factors leading to the high undernutrition levels, even when the neighbouring regions have low levels of undernutrition. It would be interesting to explore how one region failed to reduce undernutrition in high-low clusters when other regions in the neighbourhood reduced undernutrition.

As a sensitivity analysis, we applied the Getis Ord-Gi* index of spatial autocorrelation to identify clusters of undernutrition and compare it with the LISA analysis [48]. Finally, following the literature on spatial analysis [49], we used geographically weighted regression (GWR) (global and local) to highlight the differences in the relationship among variables of interest over space. We analyzed the association of a set of factors that are routinely analyzed in the empirical literature as correlates of undernutrition, including the percentage of households with electricity, median age at first sexual intercourse in years among women age 25–49 [50], percentage of live births in the five (or three) years preceding the survey delivered at a health facility [51], median duration of exclusive breastfeeding (months) [52], and percentage of women with secondary or higher education [53]. All three modules used an adaptive bi-square kernel type, and the bandwidth was chosen by minimizing the corrected Akaike information criterion. Geographically weighted regression software GWR4.0 was used for GWR analysis.

## Results

There are wide disparities in under-five child undernutrition indicators across different world regions (Table 2). South Asia has the highest prevalence of under-five child undernutrition indicators, and Europe and Central Asia have the lowest prevalence rates. The highest stunting rates are in South Asia (32.4%), closely followed by East Asia and Pacific, and Sub-Saharan Africa. The smallest stunting prevalence is in Europe and Central Asia (17.5%). Wasting prevalence is again highest in South Asia (12.6%) and lowest in Latin America and the Caribbean (1.8%). South Asia again outpaces other regions with respect to underweight (26.9%). Europe and Central Asia region has the lowest underweight prevalence rate (4.95%).

**Table 2:** Regional prevalence estimates of indicators of under-five child malnutrition

Region	Stunting	Wasting	Underweight
East Asia & Pacific	33.2	11.3	25
Europe & Central Asia	17.5	4.87	4.95
Latin America & Caribbean	19.7	1.8	6.37
Middle East & North Africa	23.6	8.22	15.5
South Asia	32.4	12.6	26.9
Sub-Saharan Africa	33.2	7.51	16.9

DHS (most recent estimates in every country)

### Stunting

**Fig. 2:**
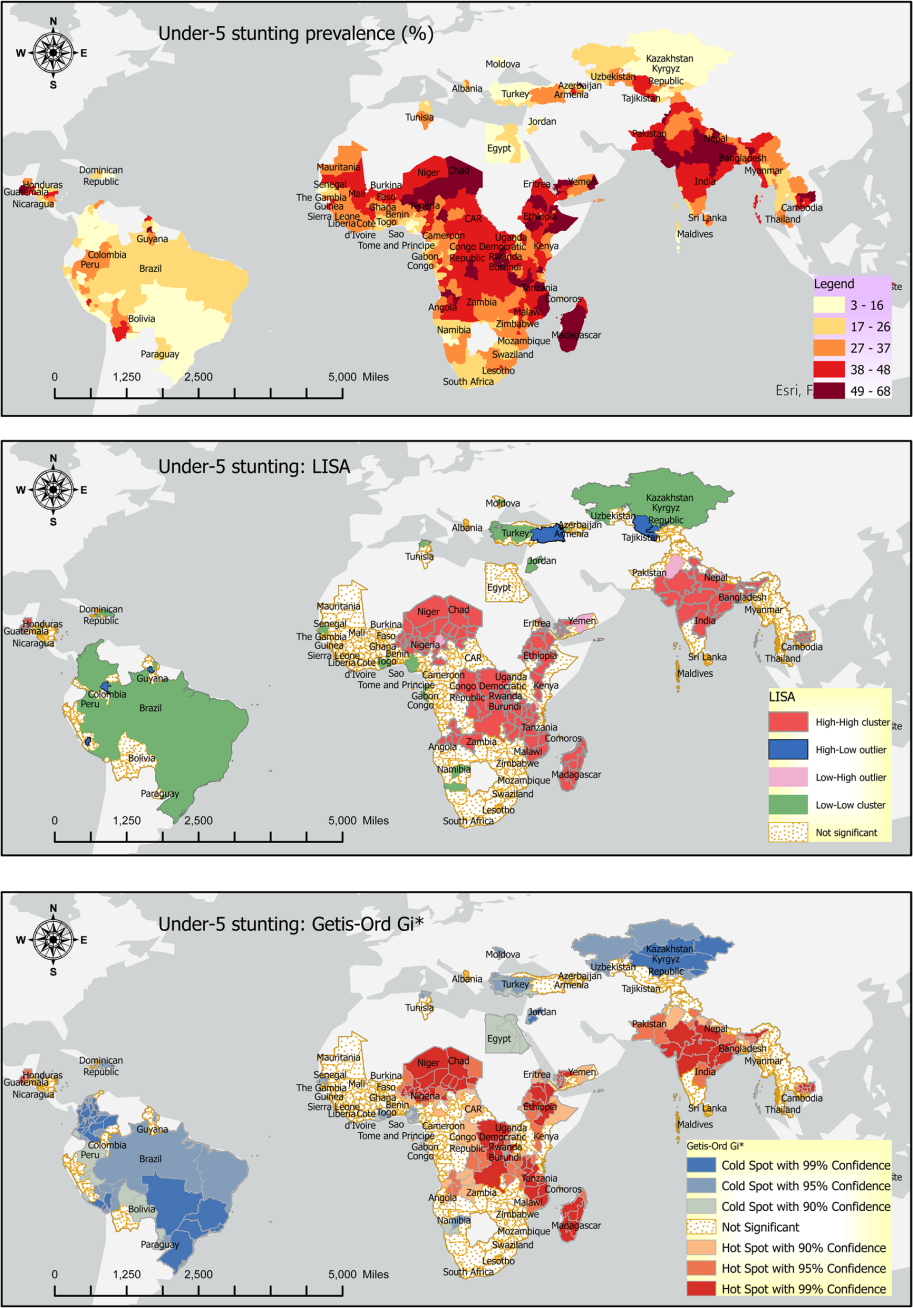
Stunting: Prevalence (top panel), LISA (middle panel), and Getis Ord-Gi* (bottom panel)

Burundi, Madagascar, Guatemala, and Yemen were the topmost countries with respect to stunting prevalence (> 46%) (Fig. 2, top panel). In contrast, Trinidad and Tobago, Dominican Republic, Jordan, and Armenia were at the bottom of countries with stunting prevalence (< 10%).

**Fig. 3:**
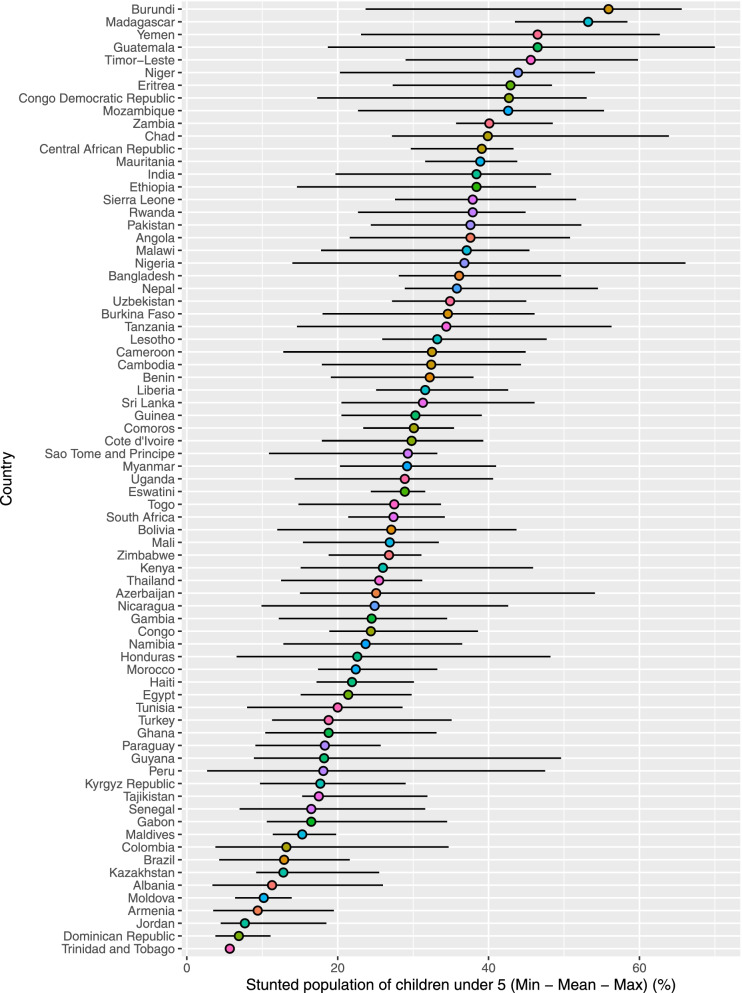
Within country disparities in the stunting rates (Source: DHS)

Around 61% of the sample countries had intra-country lowest and highest stunting rate differences of 20% percentage points or more (Fig. 3). The highest intra-country stunting differences were found in Burundi (42%), Peru (45%), Guatemala (51%), and Nigeria (52%). The lowest intra-country stunting rates differences were found in Trinidad and Tobago (0%), Swaziland (7.2%), and Dominican Republic (7.3%) Moldova (7.5%).

### Wasting

**Fig. 4:**
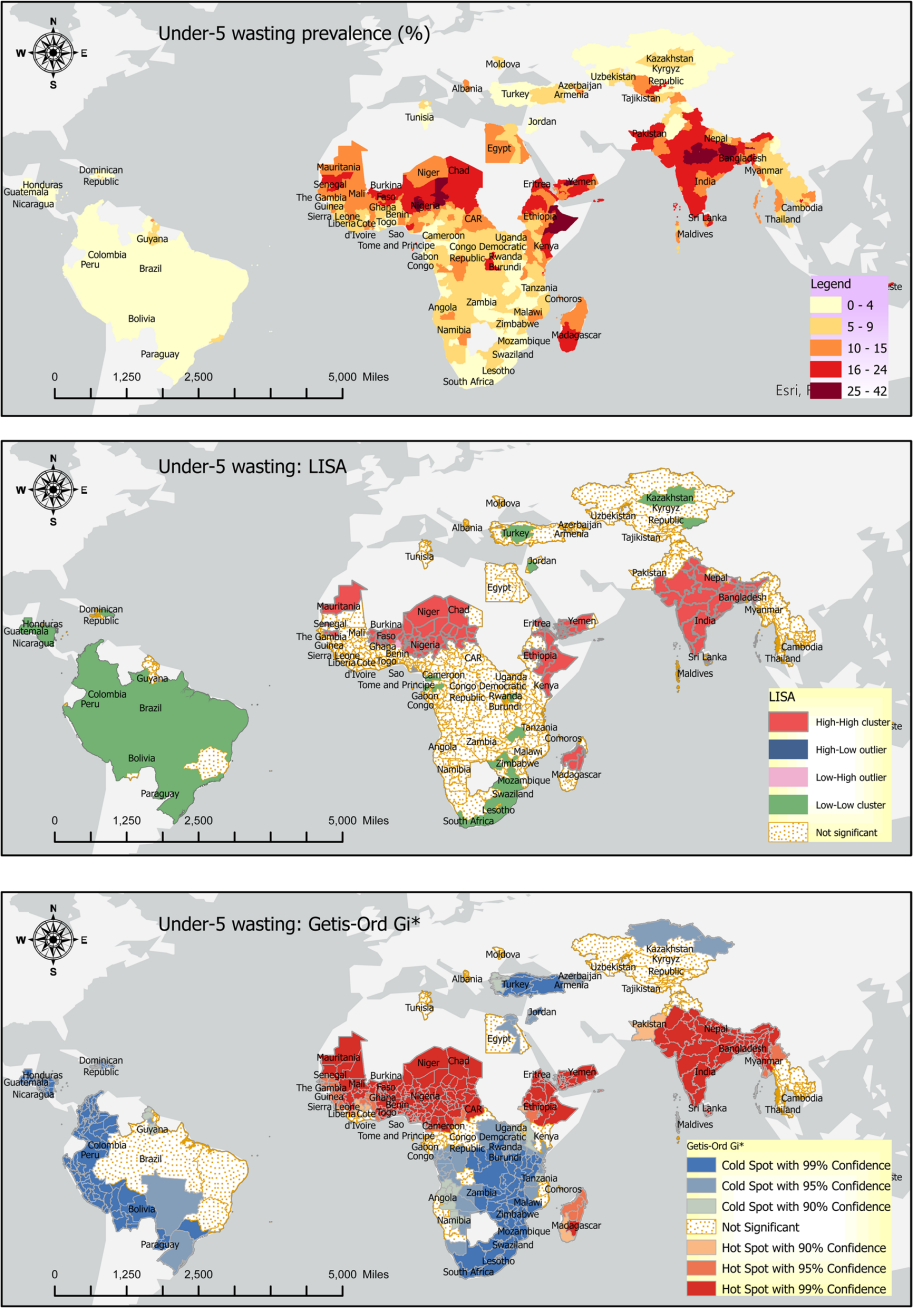
Wasting: Prevalence (top panel), LISA (middle panel), and Getis Ord-Gi* (bottom panel)

Timor-Leste, India, Niger, and Yemen had the highest wasting rates (> 16%). While Paraguay, Peru, Guatemala, Colombia, and Bolivia had the lowest wasting rates in our sample countries (< 1%) (Fig. 4: top panel).

**Fig. 5:**
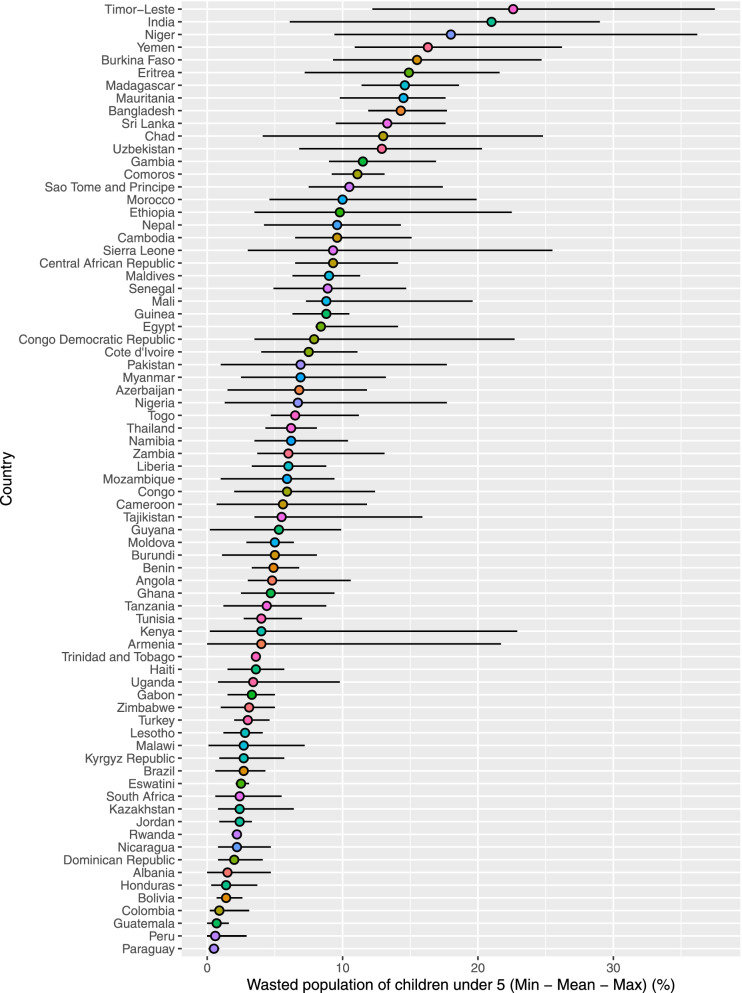
Within country disparities in the wasting rates (Source: DHS)

Around 9% of the sample countries had intra-country lowest and highest wasting rate differences of 20% percentage points or more (Fig. 5). The highest intra-country wasting differences were found in Kenya (22%), India (23%), Timor-Leste (25%), and Niger (27%). The lowest intra-country wasting rates differences were found in Trinidad and Tobago (0.0%), Rwanda (0.6%), Paraguay (0.8%) Swaziland (1.0%).

### Underweight

**Fig. 6:**
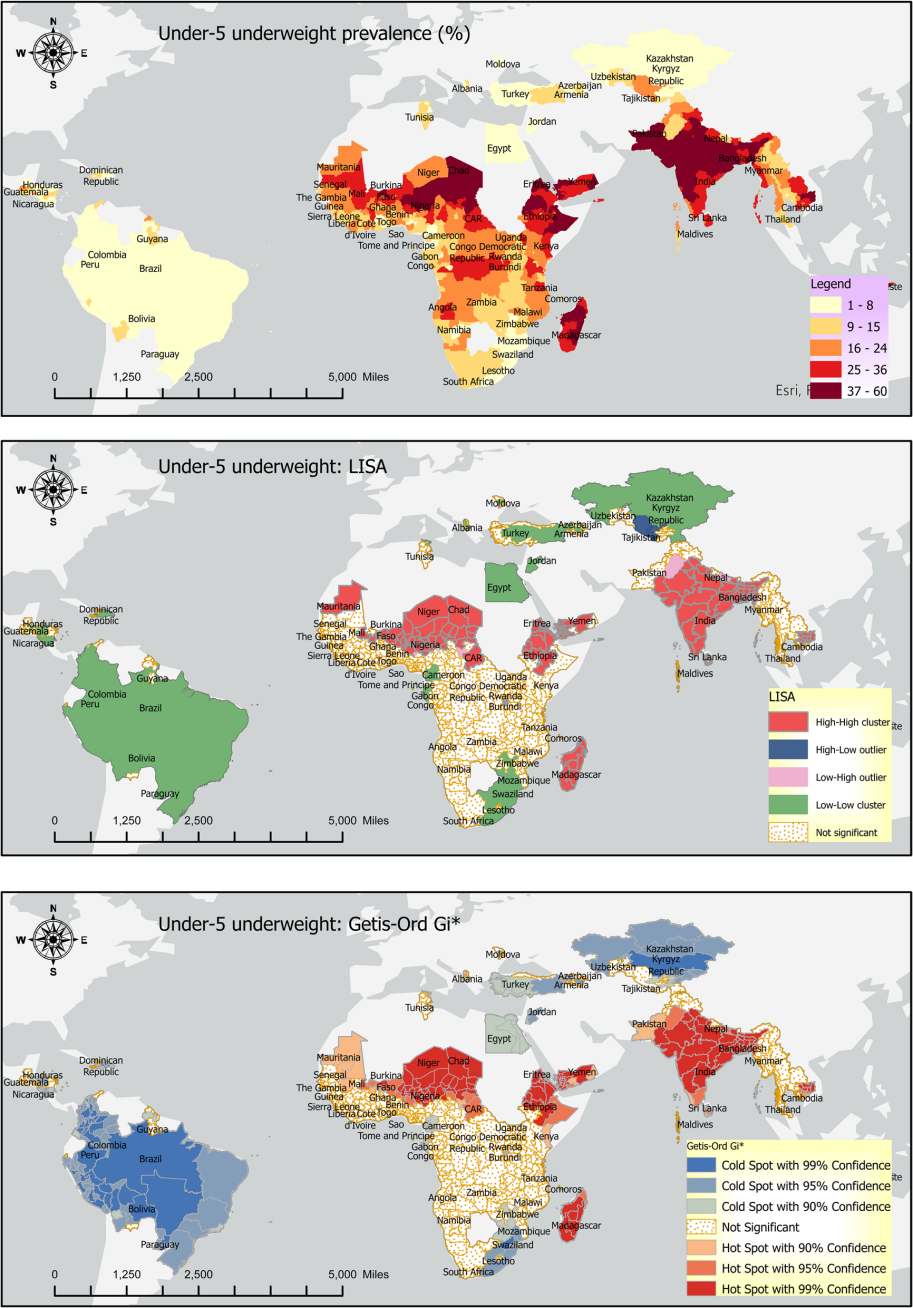
Underweight: Prevalence (top panel), LISA (middle panel), and Getis Ord-Gi* (bottom panel)

Timor-Leste, Yemen, Niger, and Madagascar had the highest underweight rates (> 36%) for the underweight nutrition indicator. In comparison, Albania, Armenia, Paraguay, Jordan, and Moldova had the smallest underweight rates (< 3.5%) (Fig. 6: top panel).

**Fig. 7:**
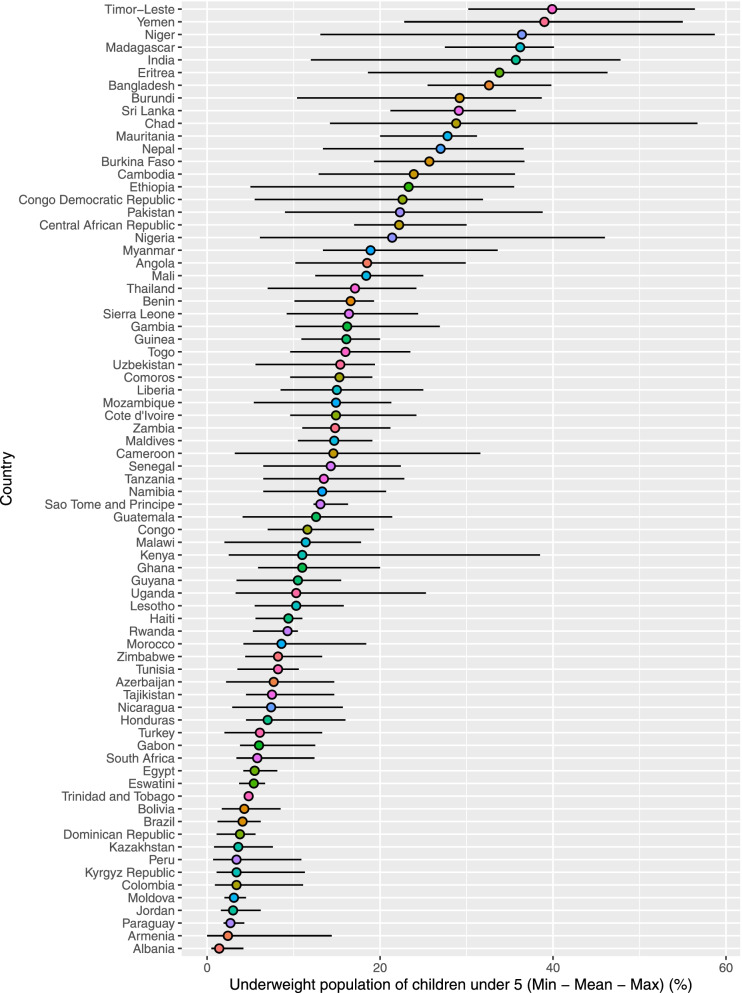
Within country disparities in the underweight rates (Source: DHS)

Around 22% of the sample countries had intra-country lowest and highest underweight rate differences of 20% percentage points or more (Fig. 7). The highest intra-country underweight differences were found in Kenya (36%), Nigeria (40%), Chad (43%), and Niger (46%). The lowest intra-country underweight rates differences were found in Trinidad and Tobago (0.0%), Paraguay (2.4%), Moldova (2.5%) Swaziland (3%).

### Stunting and wasting

Dominican Republic, Albania, Colombia, and Jordan were at the bottom of stunting and wasting indicators (least prevalence), while Madagascar, Niger, Yemen, and Timor-Leste were at the top of stunting and wasting indicators (highest prevalence).

### Stunting and underweight

Armenia, Albania, Jordan, and Moldova were at the bottom of stunting and underweight indicators (least prevalence). Madagascar, Niger, Yemen, and Timor-Leste were again at the top of stunting and underweight indicators (highest prevalence).

### Wasting and underweight

Paraguay, Albania, Peru, and Colombia were at the bottom of wasting and underweight indicators (least prevalence), while India, Niger, Yemen, and Timor-Leste were again at the top of wasting and underweight indicators (highest prevalence).

### Stunting, wasting, and underweight

All three undernutrition measures, Albania, Jordan, Colombia, and the Dominican Republic were at the bottom of the three indicators (least prevalence), while Madagascar, Niger, Yemen, and Timor-Leste were at the top of all three indicators (highest prevalence).

### Local spatial association: LISA

The Local Indicators of Spatial Association (LISA) results for the three indicators of undernutrition are provided in the middle panels of Figs. 2, 4, and 6 above. The LISA maps, alternatively called the thematic maps, are explained based on the rubric in the subsection Geospatial analysis above.

The LISA maps are of significant policy-related value because they make identifying the hotspots and cold spots easier. Additionally, the outliers, alternatively called ‘low high’ and ‘high low’ categories, are of immense policy importance because they indicate disparities in the individual indicators of undernutrition. Sometimes, the LISA maps may show disconnection from the prevalence maps; that is, the high prevalence rate in the prevalence map (Figs. 2, 4, and 6: top panels) may not be reflected in the LISA map. This is because the continuous data in the prevalence map is categorized in the LISA map. Hence, it is possible that some high-value in the prevalence map does not show up as a hotspot in the LISA map because that area is not surrounded by a significant number of other areas with a high indicator value.

### Stunting

The stunting hotspots in the sample are Bangladesh and India in South Asia, Cambodia in East Asia and Pacific, Guatemala in Latin America and the Caribbean, Yemen in the Middle East and North Africa (MENA) region, and Angola, Benin, Chad, Congo, Eritrea, Madagascar, Malawi, Mozambique, Niger, Tanzania, CAR, Ethiopia, and Rwanda in Sub-Saharan Africa (Fig. 2, middle panel). The stunting cold spots are Albania, Armenia, Kazakhstan, Kyrgyz Republic, and Moldova in Europe and Central Asia, Brazil, Colombia, Dominican Republic, Haiti, Nicaragua, Paraguay, Peru, and Guyana in Latin America and the Caribbean, Egypt and Jordan in MENA region and Cote d’Ivoire and Senegal in Sub-Saharan Africa.

Colombia, Guyana, Turkey, and Uzbekistan are high-low outliers which means that these countries have a low prevalence, but they are surrounded by high-prevalence regions. Nepal, Nigeria, Pakistan, and Yemen are low–high outliers, suggesting that these countries have low stunting prevalence, but they are surrounded by high-prevalence countries.

### Wasting

Wasting hotspots include Bangladesh and India in South Asia, Benin, Burkina Faso, Chad, Eritrea, Ethiopia, Madagascar, Mali, Mauritania, Nigeria, Senegal, and Niger in Sub-Saharan Africa and Yemen in the MENA region (Fig. 4, middle panel). Whereas wasting cold spots are Kazakhstan and the Kyrgyz Republic in Europe and Central Asia, Peru, Bolivia, Brazil, Colombia, Dominican Republic, Guatemala, Guyana, Haiti, Nicaragua, and Paraguay in Latin America and the Caribbean. Other wasting cold spots are Jordan in MENA, Sri Lanka in South Asia, Cameroon, Congo, Gabon, Lesotho, Mozambique, Rwanda, Sao Tome and Principe, South Africa, and Swaziland in sub-Saharan Africa. Nepal is the only ‘low–high’ outlier.

### Underweight

Underweight hotspots include Bangladesh, India, Sri Lanka in South Asia, Benin, Burkina Faso, Chad, Eritrea, Madagascar, Mali, Mauritania, Niger, Nigeria, CAR, and Ethiopia in sub-Saharan Africa. In East Asia and the Pacific, Cambodia and Yemen in the MENA region are also underweight hotspots (Fig. 6, middle panel). Underweight cold spots are Kazakhstan, the Kyrgyz Republic in Europe and Central Asia, Peru, Bolivia, Brazil, Colombia, Dominican Republic, Guatemala, Guyana, Haiti, Nicaragua, and Paraguay in Latin America and the Caribbean, and Jordan in the MENA region and Sri Lanka in South Asia. Other underweight cold spots are Cameroon, Congo, Gabon, Lesotho, Mozambique, Rwanda, Sao Tome and Principe, South Africa, and Swaziland. Uzbekistan is the only ‘high-low outlier’, and Pakistan is the only ‘low–high outlier’ for the underweight indicator.

### Multiple indicators

#### Hotspots (high-high clusters)

Some countries are more consistently concentrated in one LISA clustering category across multiple indicators. For example, Bangladesh and India in South Asia, Benin, Chad, Eritrea, Madagascar in Sub-Saharan Africa, and Yemen in the MENA region are hotspots of stunting and wasting. Bangladesh, India in South Asia, Cambodia in East Asia and Pacific, Central African Republic, Chad, Eritrea, Ethiopia, Madagascar, and Niger in sub-Saharan Africa, and Yemen in the MENA region are hotspots of stunting and underweight indicators. Bangladesh and India in South Asia, Benin, Burkina Faso, Chad, Eritrea, Madagascar, Mali, Mauritania, and Nigeria in Sub-Saharan Africa, and Yemen in the MENA region are hotspots of wasting and underweight. Finally, Bangladesh and India in South Asia, Benin, Chad, Eritrea, Madagascar in Sub-Saharan Africa, and Yemen in the MENA region are stunting, wasting, and underweight hotspots.

#### Cold spots (low-low clusters)

Brazil, Colombia, Dominican Republic, Haiti, Nicaragua, Paraguay, and Peru in Latin America and the Caribbean region, Jordan in the MENA region, Kazakhstan, and the Kyrgyz Republic in Europe and Central Asia are stunting and wasting cold spots. Armenia, Kazakhstan, and the Kyrgyz Republic in Europe and Central Asia, Brazil, Colombia, Dominican Republic, Haiti, Nicaragua, Paraguay, and Peru in Latin America and the Caribbean, and Egypt and Jordan in the MENA region are stunting and underweight cold spots. Peru, Bolivia, Brazil, Colombia, Dominican Republic, Guyana, Haiti, Nicaragua, Paraguay in Latin America and Caribbean, Cameroon, Congo, Gabon, Lesotho, and Swaziland in Sub-Saharan Africa, Jordan in MENA region, and Kazakhstan, Kyrgyz in Europe and Central Asia are wasting and underweight cold spots of the world. Finally, Brazil, Colombia, Dominican Republic, Haiti, Nicaragua, Paraguay, and Peru in Latin America and the Caribbean, Jordan in MENA, Kazakhstan, and the Kyrgyz Republic in Europe and Central Asia region are global cold spots of stunting, wasting, and underweight.

#### Outliers

Uzbekistan is the ‘high-low’ outlier in the stunting and underweight indicators. Nepal is the ‘low–high outlier’ in stunting and wasting indicators, and Pakistan is the ‘low–high’ indicator of stunting and underweight indicators.

### Local spatial association: Getis Ord-Gi*

Getis Ord-Gi* shows significant hotspots of high stunting rates were found in South Asia and Sub-Saharan Africa. In contrast, cold spots were found in Europe and Central Asia and Latin America, and the Caribbean (Fig. 2, bottom panel).

Getis Ord-Gi* shows significant hotspots of high wasting rates in South Asia and the northern part of Sub-Saharan Africa. At the same time, cold spots were found in southern parts of Sub-Saharan Africa, Europe and Central Asia and Latin America, and the Caribbean (Fig. 4, bottom panel).

Getis Ord-Gi* show significant hotspots of high underweight rates in South Asia and the northern part of Sub-Saharan Africa, while cold spots were found in southern parts of Sub-Saharan Africa, Europe and Central Asia, and Latin America, and the Caribbean (Fig. 6, bottom panel).

### Global spatial association

Significant positive global spatial autocorrelation exists across the study area, suggesting that adjacent countries in our analysis have similar stunting rates (Global Moran’s *I*: 0.72, *p*-value < 0.01) (Table 3). Similarly, a substantial positive global spatial autocorrelation exists across the study area, signifying that adjacent countries in our analysis have similar wasting rates (Global Moran’s *I*: 0.71, *p*-value < 0.01) and underweight rates (Global Moran’s *I*: 0.78, *p*-value < 0.01).

**Table 3:** Global spatial autocorrelation (Global Moran’s *I*)

	Global Moran’s *I*	*P*-value
Stunting	0.721525	0.000
Wasting	0.712945	0.000
Underweight	0.782262	0.000
	Geary’s *C*	*P*-value
Stunting	0.292948	0.001
Wasting	0.256921	0.001
Underweight	0.186434	0.001

Global Moran’s *I* values were computed with ArcGIS Pro 2.8.3, and Geary’s *C* values were computed with SpaceStat 4.0

The spatial autocorrelation assessed by Geary’s *C* clustering technique shows that if the *C* > 1 indicates negative spatial autocorrelation, and if the *C* < 1, it indicates positive spatial autocorrelation. Geary’s *C* also confirmed the global Moran’s *I* result in a significant geographic clustering of three measures of undernutrition. Specifically, adjacent geographical areas have similar stunting (Geary’s *C*: 0.29, *p*-value < 0.01), wasting (Geary’s *C*: 0.26, *p*-value < 0.01), and underweight (Geary’s *C*: 0.19, *p*-value < 0.01) rates.

### Geographically weighted regression

Geographically weighted regression (GWR) results suggest that an increase in the percentage of households with electricity significantly decreases stunting and increases wasting but has no significant effect on the underweight (Table 4). The local GWR models for all three measures of undernutrition have a smaller corrected AIC indicating a better model. The median coefficients of the local GWR models suggest that an increase in the percentage of households with electricity reduces stunting, wasting, and underweight.

**Table 4:** Geographically weighted regressions

		Global coefficients				Local (GWR) coefficients					
Dependent	Independent variables	Coef	SD	*t*-value	AICc	Min	LQ	Median	UQ	Max	AICc
Stunting	Intercept	59.46*	2.80	21.24	5193.66	-60.96	19.15	49.47	69.05	109.49	4715.95
Stunting	Sexual intercourse	-0.48*	0.17	-2.77		-3.62	-0.99	0.09	1.65	6.25	
Stunting	Health facility	-0.16*	0.02	-9.35		-0.71	-0.28	-0.15	-0.08	0.24	
Stunting	Exclusive breastfeeding	0.09	0.16	0.56		-2.92	-0.54	0.29	1.17	3.58	
Stunting	Women’s education	-0.11*	0.02	-4.51		-0.50	-0.25	-0.14	-0.08	0.15	
Stunting	Electricity	-0.08*	0.02	-5.01		-0.51	-0.16	-0.03	0.05	0.32	
Wasting	Intercept	9.28*	1.59	5.84	4395.13	-29.87	-5.88	5.89	21.33	62.38	3874.59
Wasting	Sexual intercourse	0.41*	0.10	4.21		-2.54	-0.86	-0.03	1.08	2.68	
Wasting	Health facility	-0.12*	0.01	-11.81		-0.31	-0.10	-0.04	0.01	0.10	
Wasting	Exclusive breastfeeding	-0.49*	0.09	-5.36		-3.45	-0.85	-0.26	-0.09	4.57	
Wasting	Women’s education	-0.04*	0.01	-2.80		-0.42	-0.06	-0.01	0.03	0.20	
Wasting	Electricity	0.02*	0.01	2.21		-0.17	-0.04	-0.02	0.02	0.43	
Underweight	Intercept	35.50*	2.50	14.19	5034.82	-56.18	3.29	15.06	46.07	124.11	4379.98
Underweight	Sexual intercourse	0.10	0.15	0.65		-4.73	-0.81	0.32	1.35	4.35	
Underweight	Health facility	-0.23*	0.02	-14.90		-0.52	-0.19	-0.10	-0.03	0.27	
Underweight	Exclusive breastfeeding	-0.36*	0.15	-2.48		-11.78	-0.95	-0.21	0.38	6.52	
Underweight	Women’s education	-0.11*	0.02	-5.07		-0.90	-0.13	-0.07	0.00	0.26	
Underweight	Electricity	0.00	0.01	0.21		-0.43	-0.11	-0.05	0.03	0.51	

^*^p < 0.05

Definitions of the independent variables is as under:

• Sexual intercourse: Median age at first sexual intercourse in years among women age 25–49

• Health facility: Percentage of live births in the five (or three) years preceding the survey delivered at a health facility

• Exclusive breastfeeding: Median duration of exclusive breastfeeding (months)

• Women’s education: Percentage of women with secondary or higher education

• Electricity: Percentage of households with electricity

*Min* Minimum, *LQ* Lower quartile, *UQ* Upper quartile, *Max* Maximum, *AICs* Corrected Akaike information criterion

An increase in the median age at first sexual intercourse among women aged 25–49 reduces stunting but counterintuitively increases wasting rates. The median coefficients of the local GWR models suggest that an increase in the median age at first sexual intercourse among women aged 25–49 reduces wasting. A higher percentage of women giving birth in a health facility is associated with lower stunting, wasting, and underweight rates. Similarly, An increase in the median duration of exclusive breastfeeding reduces wasting and underweight rates. An increase in the percentage of women with secondary or higher education significantly decreases all three undernutrition measures.

## Discussion

The geospatial distribution of the three indicators of under-five child undernutrition was investigated in this study. We found a significant within and across country variation in the stunting, wasting, and underweight rates among the under-five children’s population. The geospatial analysis also suggested that stunting, wasting, and underweight patterns show clear regional patterns. The stunting, wasting, and underweight hotspots in the sample were mostly situated in South Asia and Sub-Saharan Africa. The stunting, wasting, and underweight cold spots were primarily concentrated in Europe and Central Asia and the Latin America, and the Caribbean regions.

Some countries are more consistently concentrated in one LISA clustering category across multiple indicators. Bangladesh and India in South Asia, Benin, Chad, Eritrea, and Madagascar in Sub-Saharan Africa, and Yemen in the MENA region are stunting, wasting, and underweight hotspots. Brazil, Colombia, Dominican Republic, Haiti, Nicaragua, Paraguay, and Peru in Latin America and the Caribbean, Jordan in MENA and Kazakhstan, and the Kyrgyz Republic in Europe and Central Asia are global stunting, wasting, and underweight cold spots.

An exploration of the spatial distribution of key health indicators helps identify the patterns of undernutrition and identify the areas where the prevalence of undernutrition is extraordinarily high. The geospatial analysis also identifies the areas with extreme inequalities. A cross-country geospatial analysis could enable policymakers to tailor coordinated policies for better results.

While it is crucial to identify the determinants of child undernutrition, the geographic location has not been analyzed in detail as a correlate of child undernutrition. Health inequalities can be observed by the patterns of health outcomes [54, 55]. Sometimes health inequality is analyzed as a function of the wealth status [56, 57]. Nevertheless, large cross-country clusters of undernutrition indicators may reflect comparable life circumstances. Cultures often transcend national boundaries. Historically, many homogenous ethnic groups were divided into independent nation-states at the end of the colonial system. Consequently, population groups in the neighboring areas share many cultural traits, even though they are in different independent countries [58]. Since child undernutrition indicators, such as stunting, wasting, and underweight, are both a function of food intake and genetic and hereditary characteristics, it is plausible to see similar patterns within and across countries.

The hotspots and cold spots dominate the undernutrition patterns in the developing world, with very few outliers with ‘high low’ clusters or ‘low high’ clusters. However, the wide disparities in child undernutrition within the countries may indicate the varying levels of quality and quantity of government health interventions or the differential impact of the government interventions on different population sub-groups.

Existing evidence shows that stunting is closely associated with culturally distinct food consumption patterns [59]. Early initiation of breastfeeding is extremely low in LMICs. According to the recent global trends, less than 50%, of children receive early initiation of breastfeeding, only about 40% are exclusively breastfed in the first six months, and around 33% children do not continue breastfeeding in the second year of their life.^1^ Evidence suggests that early initiation of breastfeeding significantly reduces stunting and other types of undernutrition [60]. Exclusive breastfeeding also has a protective effect against the risk of child undernutrition [52].

A balanced diet, with an adequate intake of fruit and vegetables and poultry products, is not accessible for most people in LMICs. Evidence suggests that complementary feeding (consumption of solid, semi-solid, or soft foods in addition to breastmilk after six months of birth) has also a significant role in preventing undernutrition [61–63]. However, to realize full health benefits, complementary feeding must be initiated in the sixth month of a child’s life [64]. Complementary food must also satisfy minimum dietary diversity [65]. Standards of minimum dietary diversity are satisfied when a child is fed from at least four out of seven food groups, including i) grains, roots, and tubers, ii) legumes and nuts, iii) dairy products, iv) flesh foods, v) eggs, vi) vitamin-A rich fruits and vegetables, and vii) other fruits and vegetables [66]. Additionally, complementary food should follow recommended intake consistent with the child’s age group. For example, recommended intake of iron-rich foods such as meats, poultry, and fortified infant cereals is different for 6–9 months children and 9–12 months old children [67]. A study in Malawi concludes that despite the availability of crops that make up major components of complementary food, the consumption of complementary food remains low due to cultural traditions and limited awareness of the health benefits of complementary food [68]. There has been a growing shift in food consumption patterns favoring readily available processed food [69]. Food insecurity is a critical problem in the developing world exacerbated by the violent swings in climatic patterns [70]. But an even pressing issue is the inequitable distribution of food and other productive resources, which subjects some sections of the society to poverty [71].

Developing countries generally have a low productive capacity, which barely leaves enough funds for productive investment [72]. Consequently, they can hardly go beyond satisfying their basic needs. Since the per capita income is very little, the governments cannot raise sufficient revenue through taxes and cannot put the economy on a higher growth trajectory. Consequently, governments have insufficient funds to adequately invest in the health and education sector to promote human capital, and such countries have to labour under enormous foreign debts. The aid and grants and official development assistance (ODA) often come with strings attached. Therefore, there is not much space for maneuvering for the governments in LMICs.

Maternal health also contributes significantly to the nutritional profile of the children [73]. Usually, the young mothers are not adequately developed physically to feed their children. Additionally, women in the developing world often have a lower status within the household. They have limited autonomy in the decision-making process related to their children’s health. In some cultures, women are not allowed to venture out to receive medical care without a male relative. They often are discriminated against concerning the distribution of food within the household. Some are subjected to domestic violence, with profound health implications for themselves and their children. A combination of all these factors contributes to inadequate food intake, which thereby adversely affects the development of the fetus, leading to child undernutrition in the long term.

The average height of the mother is also a significant predictor of stunting [74]. With an average maternal height in LMICs significantly less than the average maternal height in the developed world, stunting can sometimes be explained in terms of genetic characteristics. Apart from the genetic characteristics, some other factors that play a role in explaining the intergenerational transmission of undernutrition are the intergenerational transmission of poverty [75]. From the point of view of human evolution, women have smaller pelvises than other higher primates, and stunted women have even smaller pelvises, which significantly increase the risk of child mortality during birth to a child with a large head circumference [75]. Because of the known risks of giving birth to a large baby, many women in the different parts of the world “eat down” during pregnancy, increasing the risk of child undernutrition [75].

A substantial disease burden in LMICs can be attributed to the health conditions generally addressed in the developed world. For example, malaria, contained mainly in the developed world, is a major killer in LMICs [76]. Furthermore, chronic diarrhoeal diseases and other infectious diseases, some of which are avoidable through effective vaccination, also contribute to the inaccessibility of a balanced diet and, in turn, lead to stunting. Also, HIV AIDS in Africa poses a significant disease burden on the population [77]. Similarly, South Asia also has a fragile public healthcare system, which cannot effectively satisfy a massive population’s health needs. Therefore, the disease burden can translate into substantial out-of-pocket expenses in many households and lead to child undernutrition [78].

### Priorities for regions with high nutritional inequalities

We find that South Asia has the highest prevalence of under-five child undernutrition. This emphasizes the need to prioritize children’s nutritional intake in South Asia and understand why South Asia is leading the world in this area. The stunting and wasting hotspots in the sample are Bangladesh and India in South Asia, while for under-weight, Sri Lanka is also included besides Bangladesh and India. This study thus identifies that these three countries require immediate priorities by the national governments of the respective countries and international organizations to identify the causes of undernutrition to help these countries come out of this situation. A closer look at these countries illustrates the problems in the health service delivery of these countries and problems such as maternal health, food consumption patterns, food insecurity, lack of safe drinking water, conflicts, and so on [79]. To tackle these challenges, it is also proposed that a new South Asian Health Organization is formed where these countries tackle common challenges together and make concerted efforts to tackle under-five child undernutrition and track their progress over time.

Currently, many of the neighboring countries in the developing world have regional conflicts, and therefore a coordinated strategy to fight child undernutrition on a regional basis seems highly challenging. The decades-long enmity between nuclear powers like India and Pakistan in South Asia is one such example of hatred among the neighbors. Similarly, many countries in sub-Saharan Africa have been the sworn enemies of their neighbors and have remained engaged in bloody conflicts. While the national policies have to address the within-country nutritional inequalities, any global initiative to alleviate child undernutrition may need to keep such decades-long political conflicts or any related external factors into account for successful policy interventions. Since maternal health also contributes significantly to the children’s nutrition [73], the governments in the respective countries need to significantly increase investments in maternal health, which will help improve the nutritional status of the children.

The correlates of child undernutrition used in the geographically weighted regressions corroborate the existing evidence. Exclusive breastfeeding has a protective effect on child undernutrition [52], and child marriages increase the risk of child undernutrition [50]. Maternal education protects against child malnutrition [53]. Relative to home-based delivery, the birth of a child at the health facility reduces the risk of child undernutrition [51].

## Limitation

Previously, undernutrition and overnutrition were generally analyzed as two distinct domains based on the assumption that these two nutritional statuses are fundamentally different, pose different challenges, affect different populations, and have different risk factors [80]. However, in recent years, a new understanding has emerged that two types of malnutrition can co-occur within communities, families, and even individuals and is aptly called the “double burden of malnutrition.” It is argued that both undernutrition and overnutrition share common drivers, including inadequate early life nutrition, dietary diversity, and socioeconomic factors [81]. Similarities in the risk factors of both nutritional issues require a uniform measure to address both problems. A double-duty action seeks to prevent the risk of undernutrition and overnutrition simultaneously. However, we have used three measures of undernutrition because the georeferenced data necessary for spatial analysis was available in case of stunting, wasting, and underweight. So restricting the study to include only the undernutrition variables is a significant limitation of our study. As we analyzed observational data, establishing a casual relationship is not possible.

One limitation of our research was the non-existence of panel data to assess the geography of nutritional inequalities over time. Future research could focus on cross-country panel data studies to assess such inequalities to understand which countries are faring better in improving under-five child nutrition and the underlying reasons for the improvement. Another interesting direction for future research would be to look at nutritional inequalities among children in different age groups.

## Conclusion

We analysed geospatial distribution of the three indicators of under-five child undernutrition. We found a significant within and across country variation in stunting, wasting, and underweight rates. The geospatial analysis suggested that three measures of child undernutrition showed clear regional patterns. Child undernutrition hotspots in the sample were mostly in South Asia and Sub-Saharan Africa, and cold spots were mostly concentrated in Europe, Central Asia, Latin America and the Caribbean.

## Data Availability

DHS data set is publically available online on the following link: https://dhsprogram.com/data/available-datasets.cfm

## Footnotes

1
https://data.unicef.org/resources/world-breastfeeding-week-2020/
